# Supplementary material for: HGTector: an automated method facilitating genome-wide discovery of putative horizontal gene transfers
Source: BMC Genomics. 2014 Aug 26;15(1):717. doi: 10.1186/1471-2164-15-717 (PMC4155097; doi:10.1186/1471-2164-15-717)
Supplement: Supplementary file 1 — Additional file 1: Figure S1: Figure S1. Illustration of patterns of BLAST hit distribution and possible explanations. Figure S2. Precision-recall plot of results on simulated genomes. Figure S3. Relationship between global HGT rate and performance of methods. Figure S4. Fingerprints of genomes of various organisms. Figure S5. Distribution of BLAST hit weights of the R. felis genome. Figure S6. Heat map indicating percentages of predicted HGT-derived genes by putative bacterial donor groups in Rickettsia genomes. Figure S7. Heat map indicating percentages of predicted HGT-derived genes by functional annotations in Rickettsia genomes. Figure S8. Stability of results on the Rickettsia dataset with various simulated stochastic events. Figure S9. Stability of results on the Rickettsia dataset with simulated taxon sampling bias. Figure S10. Stability of results on the Rickettsia dataset with smaller sample size of genes for fingerprint calculation. Figure S11. Comparison of prediction results in the R. felis genome by multiple methods. (PDF 4 MB) [file 12864_2014_6395_MOESM1_ESM.pdf]

Supplementary Figures for Manuscript:

**HGTector: An automated method facilitating genome-wide discovery of putative horizontal gene transfers**

Authors: Qiyun Zhu, Michael Kosoy, Katharina Dittmar

# Contents

**Figure S1** Illustration of patterns of BLAST hit distribution and possible explanations.

**Figure S2** Precision-recall plot of results on simulated genomes.

**Figure S3** Relationship between global HGT rate and performance of methods.

**Figure S4** Fingerprints of genomes of various organisms.

**Figure S5** Distribution of BLAST hit weights of the *R. felis* genome.

**Figure S6** Heat map indicating percentages of predicted HGT-derived genes by putative bacterial donor groups in *Rickettsia* genomes.

**Figure S7** Heat map indicating percentages of predicted HGT-derived genes by functional annotations in *Rickettsia* genomes.

**Figure S8** Stability of results on the *Rickettsia* dataset with various simulated stochastic events.

**Figure S9** Stability of results on the *Rickettsia* dataset with simulated taxon sampling bias.

**Figure S10** Stability of results on the *Rickettsia* dataset with smaller sample size of genes for fingerprint calculation.

**Figure S11** Comparison of prediction results in the *R. felis* genome by multiple methods.

**A.** Normal

| Match | Bit score |
|---|---|
| self0 | 100 |
| self1 | 98 |
| self2 | 97 |
| self3 | 94 |
| self4 | 92 |
| close1 | 85 |
| close2 | 84 |
| close3 | 83 |
| close4 | 81 |
| close5 | 81 |
| close6 | 80 |
| distal1 | 75 |
| distal2 | 72 |
| distal3 | 71 |
| distal4 | 68 |
| distal5 | 66 |
| distal6 | 62 |
| distal7 | 61 |
| distal8 | 59 |
| ... | |

self + close + distal + HGT: no

**B.** HGT (recent)

| Match | Bit score |
|---|---|
| self0 | 100 |
| distal1 | 99 |
| distal2 | 98 |
| distal3 | 97 |
| distal4 | 95 |
| distal5 | 92 |
| distal6 | 90 |
| distal7 | 89 |
| distal8 | 88 |
| distal9 | 85 |
| distal10 | 82 |
| distal11 | 79 |
| distal12 | 78 |
| distal13 | 77 |
| distal14 | 76 |
| distal15 | 73 |
| distal16 | 70 |
| distal17 | 67 |
| distal18 | 64 |
| ... | |

self - close - distal + HGT: yes

**C.** HGT (ancient)

| Match | Bit score |
|---|---|
| self0 | 100 |
| self1 | 98 |
| self2 | 97 |
| self3 | 94 |
| distal1 | 90 |
| distal2 | 88 |
| distal3 | 87 |
| distal4 | 85 |
| distal5 | 83 |
| distal6 | 82 |
| distal7 | 80 |
| distal8 | 79 |
| distal9 | 76 |
| distal10 | 72 |
| distal11 | 70 |
| distal12 | 67 |
| distal13 | 66 |
| distal14 | 62 |
| distal15 | 58 |
| ... | |

self + close - distal + HGT: yes

**D.** HGT + paralogy

| Match | Bit score |
|---|---|
| self0 | 100 |
| self1 | 98 |
| self2 | 97 |
| self3 | 94 |
| distal1 | 90 |
| distal2 | 89 |
| distal3 | 87 |
| close1 | 86 |
| distal4 | 85 |
| distal5 | 83 |
| distal6 | 80 |
| distal7 | 77 |
| distal8 | 74 |
| distal9 | 72 |
| distal10 | 70 |
| distal11 | 64 |
| close2 | 57 |
| distal12 | 53 |
| distal13 | 51 |
| ... | |

self + close - distal + HGT: yes

**E.** Gene loss in close lineages

| Match | Bit score |
|---|---|
| self0 | 100 |
| self1 | 98 |
| self2 | 97 |
| self3 | 94 |
| self4 | 92 |
| close1 | 85 |
| close2 | 84 |
| close3 | 83 |
| close4 | 81 |
| distal1 | 75 |
| distal2 | 72 |
| distal3 | 71 |
| distal4 | 68 |
| distal5 | 66 |
| distal6 | 62 |
| distal7 | 61 |
| distal8 | 59 |
| distal9 | 55 |
| distal10 | 48 |
| ... | |

self + close + distal + HGT: no

**F.** Outgoing HGT / contamination

| Match | Bit score |
|---|---|
| self0 | 100 |
| distal1 | 98 |
| self1 | 97 |
| self2 | 96 |
| self3 | 94 |
| self4 | 92 |
| close1 | 85 |
| close2 | 84 |
| close3 | 83 |
| close4 | 81 |
| close5 | 81 |
| close6 | 80 |
| distal2 | 72 |
| distal3 | 71 |
| distal4 | 68 |
| distal5 | 66 |
| distal6 | 62 |
| distal7 | 61 |
| distal8 | 59 |
| ... | |

self + close + distal + HGT: no

**G.** ORFan / annotation error

| Match | Bit score |
|---|---|
| self0 | 100 |
| self1 | 98 |
| self2 | 97 |
| self3 | 96 |
| self4 | 95 |

self + close - distal - HGT: no

or

| Match | Bit score |
|---|---|
| self0 | 100 |
| self1 | 98 |
| self2 | 97 |
| self3 | 96 |
| distal1 | 43 |
| distal2 | 40 |

self + close - distal - HGT: no

**H.** Gene loss in self lineages

| Match | Bit score |
|---|---|
| self0 | 100 |
| self1 | 98 |
| close1 | 85 |
| close2 | 84 |
| close3 | 83 |
| close4 | 81 |
| close5 | 81 |
| close6 | 80 |
| distal1 | 75 |
| distal2 | 72 |
| distal3 | 71 |
| distal4 | 68 |
| distal5 | 66 |
| distal6 | 62 |
| distal7 | 61 |
| distal8 | 59 |
| distal9 | 56 |
| distal10 | 50 |
| distal11 | 47 |
| ... | |

self - close + distal + HGT: no

Figure S1 Illustration of patterns of BLAST hit distribution and possible explanations.

Each panel represents a hypothetical set of match organism names and bit scores. “-” (atypical) or “+” (typical) at the bottom of each panel, indicates status of weight distribution in each hierarchical category (*self*, *close* and *distal*). The prediction result is indicated by “yes” (HGT-derived) or “no” (not HGT-derived) after the status of the weights. A brief description of possible explanations of the gene's evolutionary history is indicated as the title of each panel. **(A)** A normal gene with a typical vertical inheritance history. **(B)** *Close* hits and *self* hits (except for the query) are absent, suggesting HGT from a *distal* organism to the query species or its recent ancestor. **(C)** *Close* hits are absent, suggesting HGT from a *distal* organism to the common ancestor of *self0* to *self*3. **(D)** One or a few *close* hits are present but the weight of the *close* group is below cutoff, suggesting HGT, and the presence of *close* hits might be due to paralogy or multiple transfer events. **(E)** A few *close* hits are absent but the overall *close* weight is normal, suggesting vertical inheritance, but loss of orthologs in some of the *close* lineages. **(F)** The overall pattern is typical, except that one or a few *distal* hits have high bit scores. This may represent HGT events from *self* to the *distal* group (outgoing HGT). It may also be caused by database error. **(G)** There are no or few *close* and *distal* hits, suggesting that the gene may be an ORFan derived from de novo gene origination or HGT from an unsequenced group of organisms, or simply genome annotation error. **(H)** The *close* weight is typical but the *self* weight is atypical, suggesting the gene might be lost in a considerable portion of the *self* group.

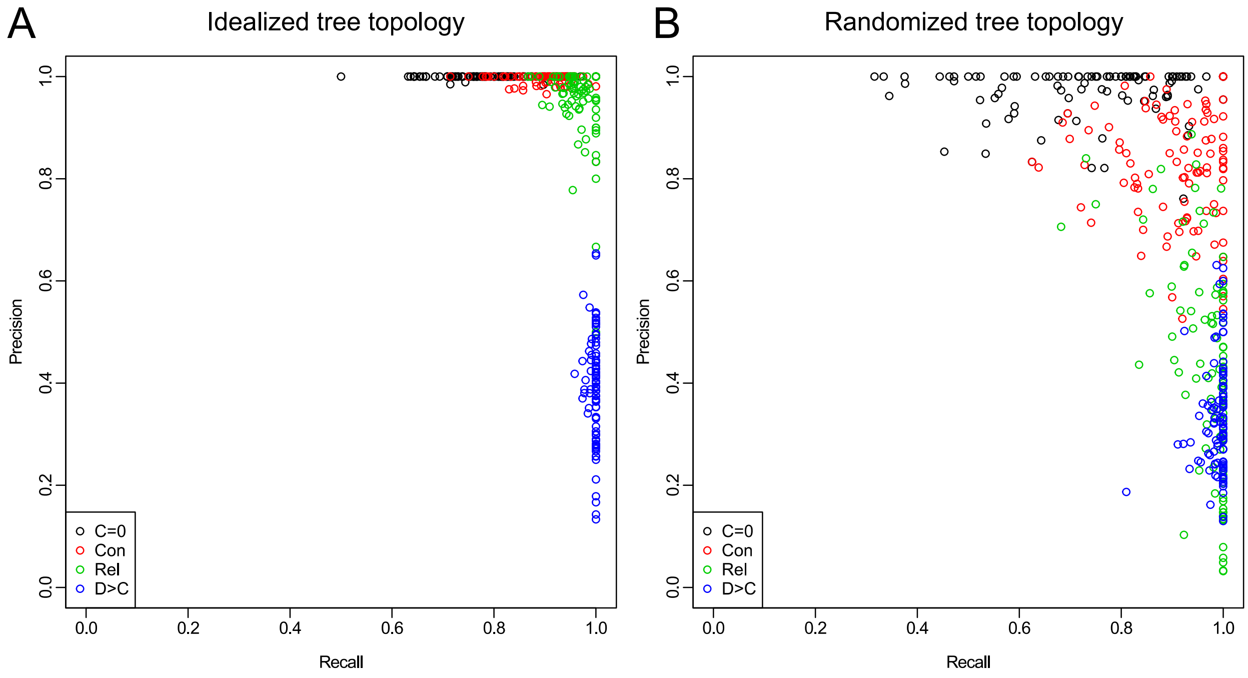


## Figure S2 Precision-recall plot of results on simulated genomes.

Each panel contains the results from 100 tests. “Con” and “Rel” represent conservative and relaxed criteria of choosing cutoff in HGTector analysis. “C=0” and “D>C” are two criteria under conventional BLAST-based method.

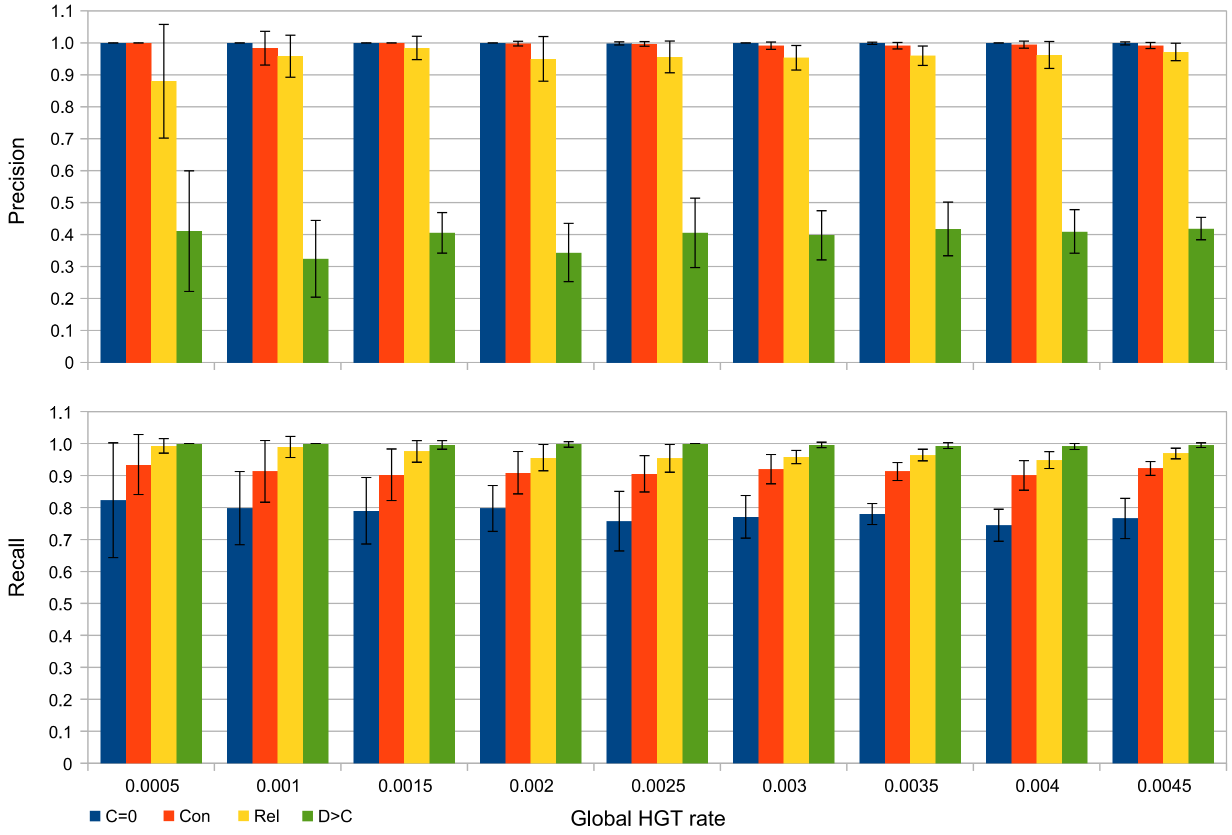


### Figure S3 Relationship between global HGT rate and performance of methods

The performance under the idealized topology was plotted against incremental global HGT rates (unit: frequency of events per gene per time unit). Each bin contains ten replicates. “Con” and “Rel” represent conservative and relaxed criteria of choosing cutoff in HGTector analysis. “C=0” and “D>C” are two criteria under conventional BLAST-based method. Error bars represent standard deviations.

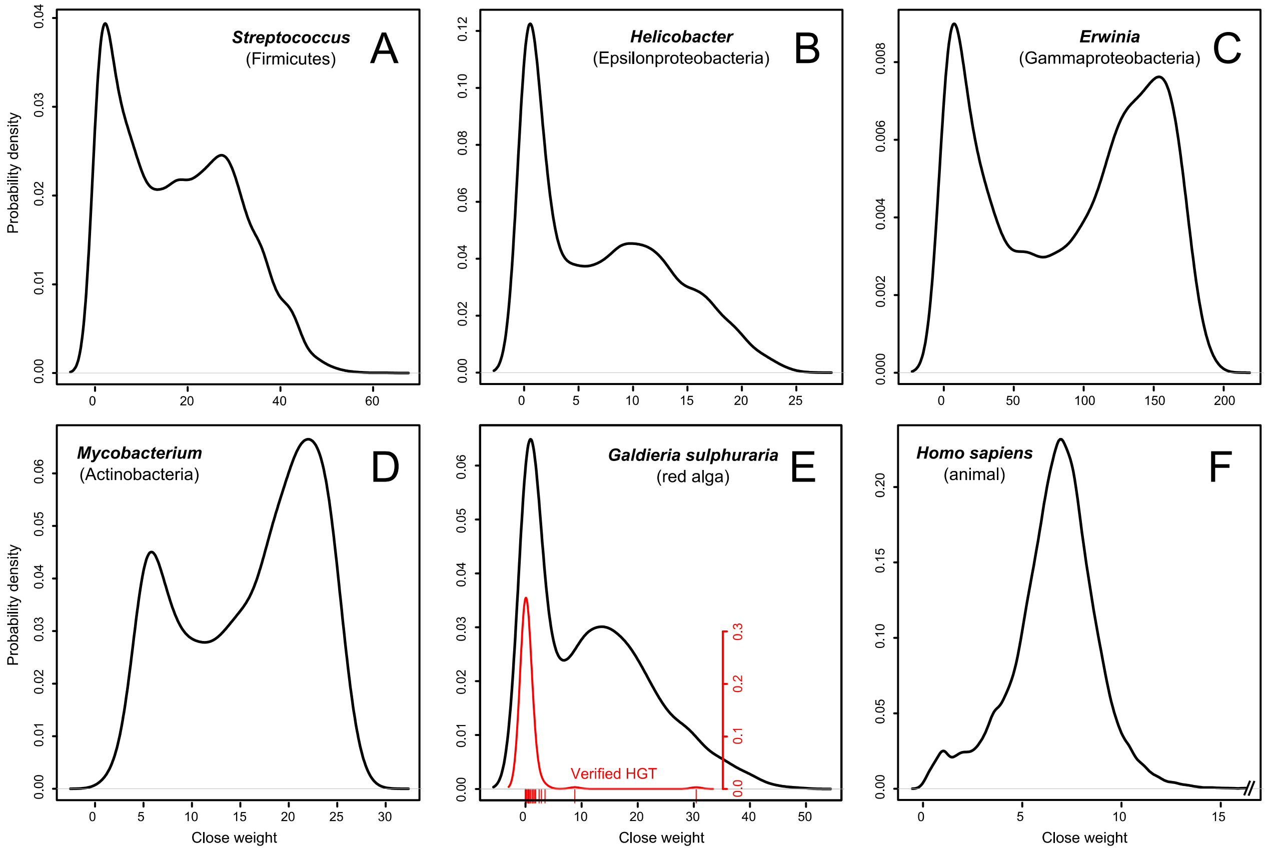


Figure S4 Fingerprints of genomes of various organisms.

The *close* weight distributions computed on multiple real genomic datasets are plotted as kernel density functions. A taxon name that best describes the *self* group is labeled in each panel. In panel **E** (*Galdieria sulphuraria*), the red curve and rug represent the HGT-derived genes identified by Schonknecht et al. (2013) using a phylogenetic approach.

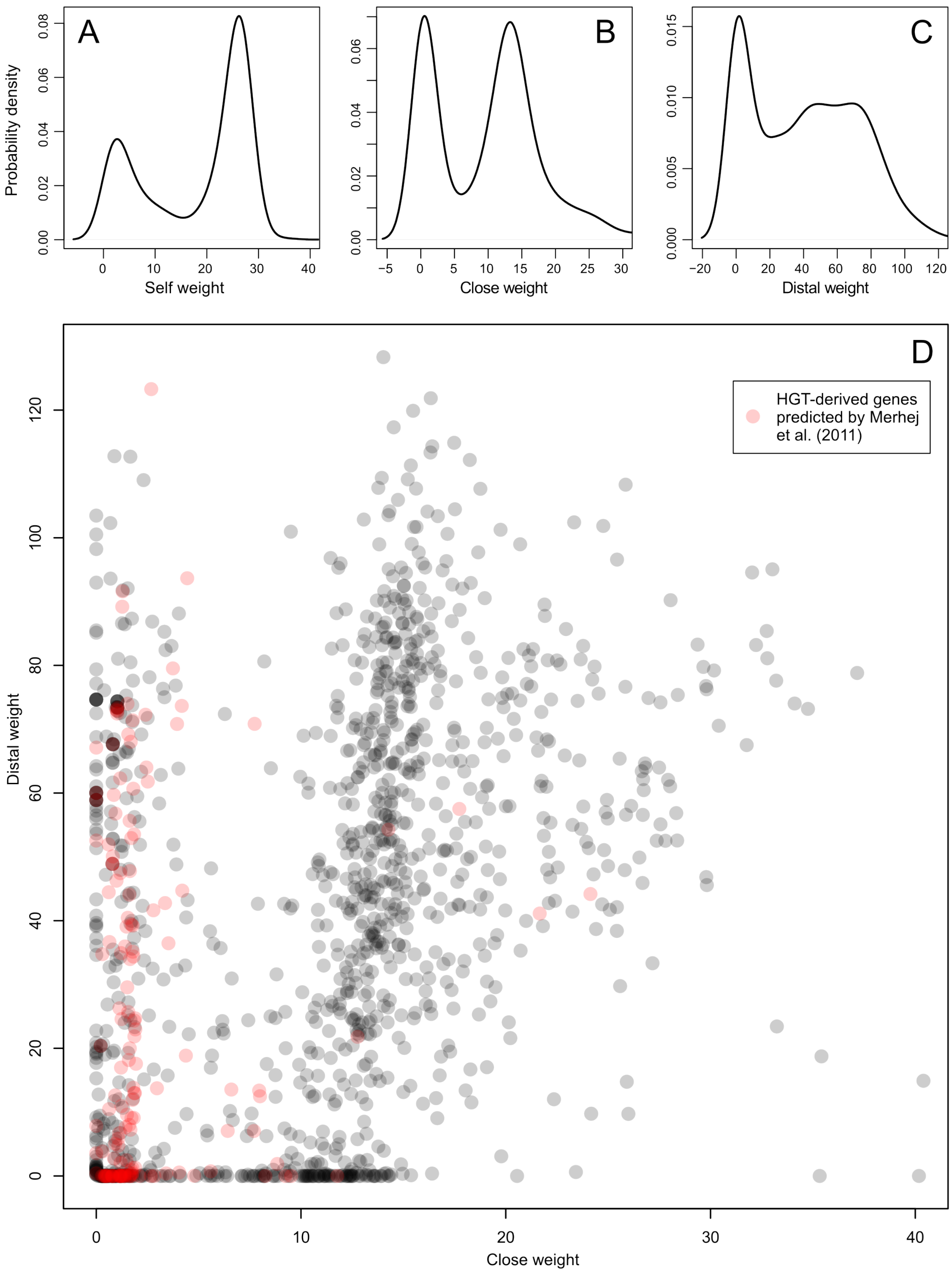


Figure S5 Distribution of BLAST hit weights of the *R. felis* genome.

BLAST hit weights of all 1400 protein-coding genes in the *R. felis* genome are plotted. (**A-C**) Kernel density functions of the *self*, *close* and *distal* weights. The x-axis represents the weight of each gene. The y-axis represents the probability density of genes with the corresponding weight in the genomes. (**D**) A scatter plot of the *distal* weight against the *close* weight. Each dot represents one gene. Genes predicted to be HGT-derived by Merhej et al. (2011) using a phylogenetic approach are colored red.

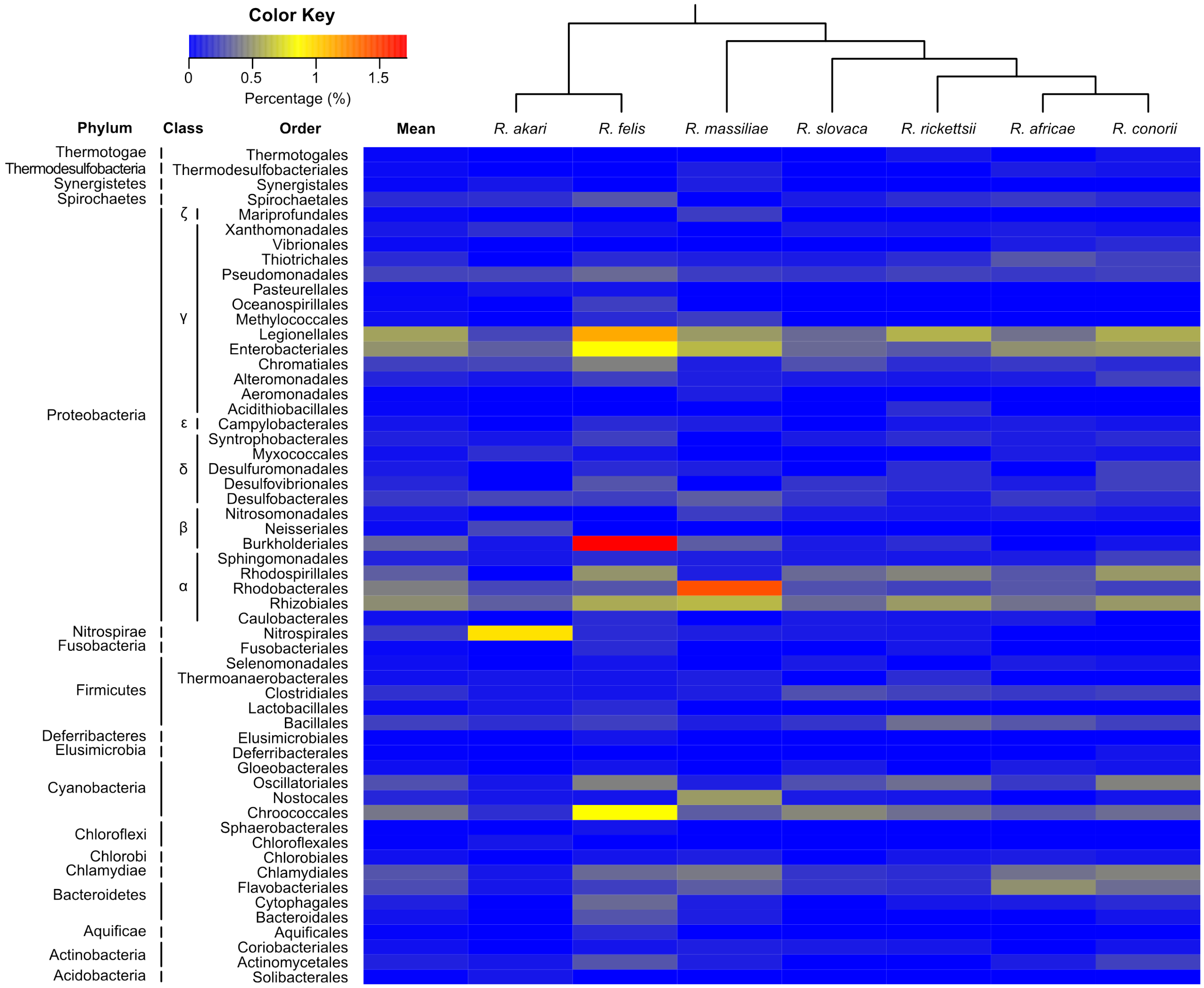


Figure S6 Heat map indicating percentages of predicted HGT-derived genes by putative bacterial donor groups in *Rickettsia* genomes.

Percentages were calculated by dividing the number of HGT-derived genes per putative donor order by the total number of protein-coding genes of a genome. The phylogeny of the *Rickettsia* species following Merhej et al. (2011) is indicated in the header.

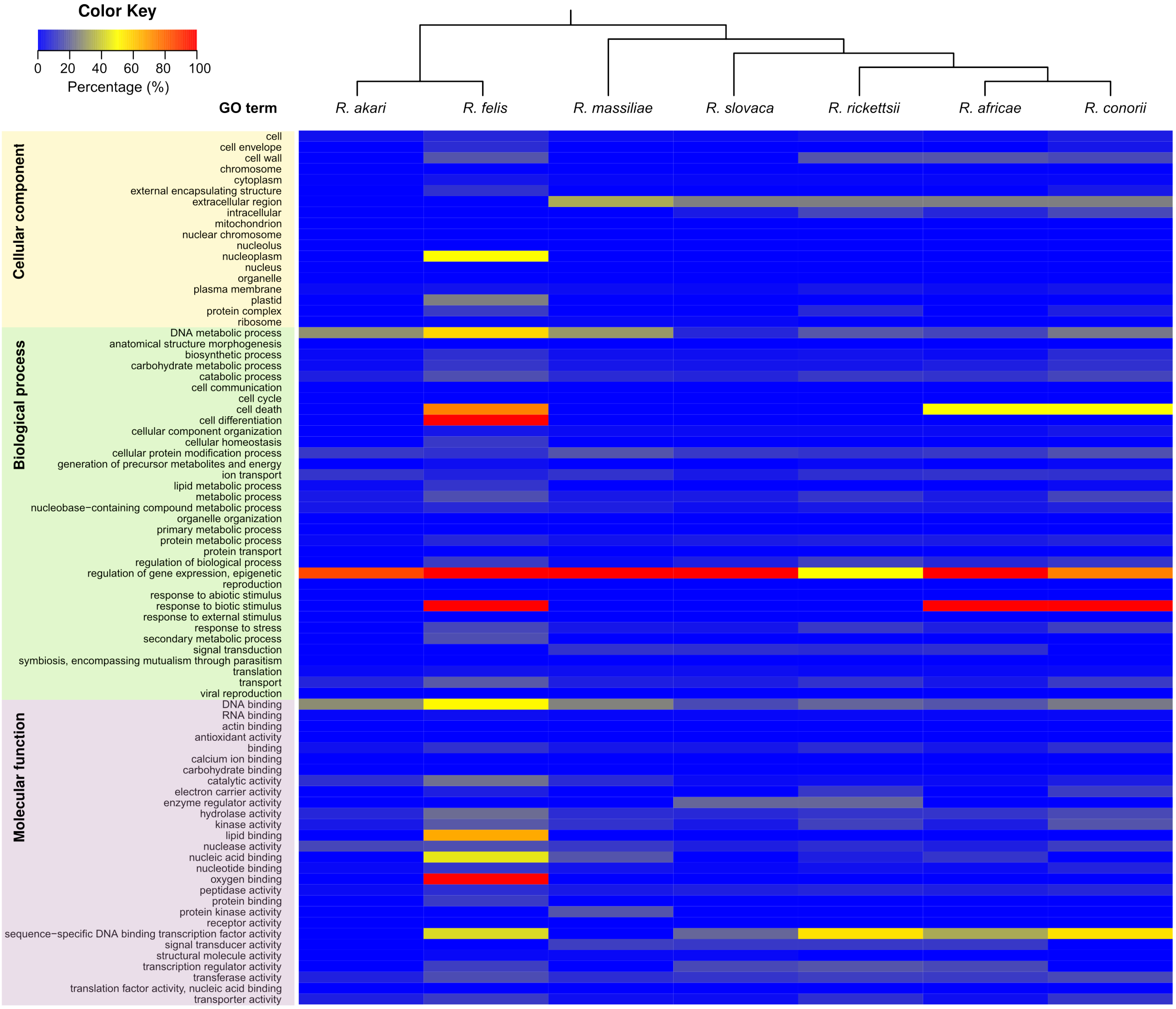


Figure S7 Heat map indicating percentages of predicted HGT-derived genes by functional annotations in *Rickettsia* genomes.

Genes were annotated using Gene Ontology (GO) and refined by generic GO slim. Proportions were calculated by dividing the number of HGT-derived genes associated with a GO by the total number of genes associated with this GO. The phylogeny of the *Rickettsia* species is indicated in the header.

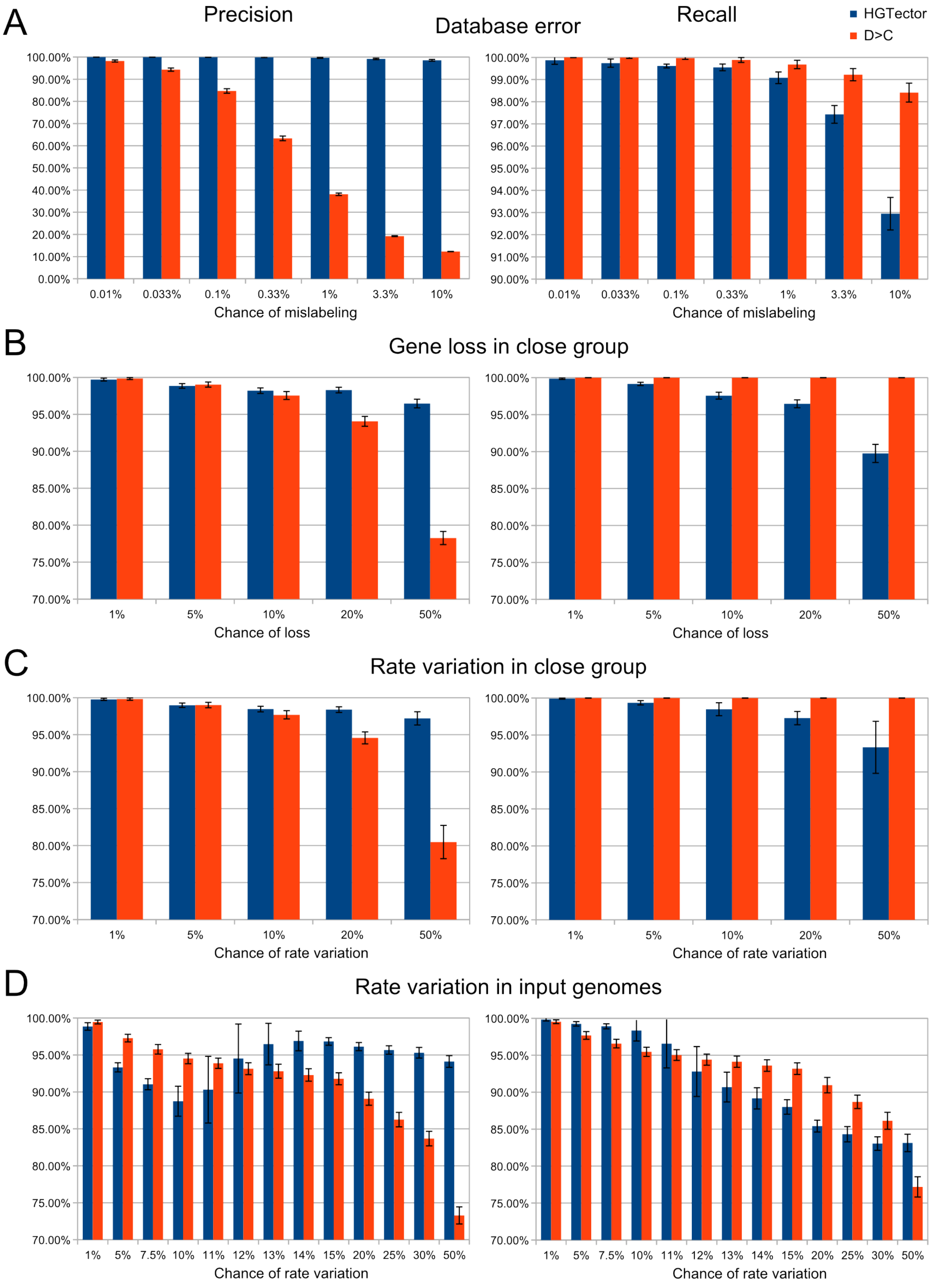


Figure S8 Stability of results on the *Rickettsia* dataset with various simulated stochastic events.

Results of HGTector using the conservative criterion (red) and the conventional BLAST best match approach using the D>C criterion (blue) were cross-compared. Precision (left panels) and recall (right panels) were computed with the standard result as the reference. Each group has 100 replicates. Error bars indicate standard deviations.

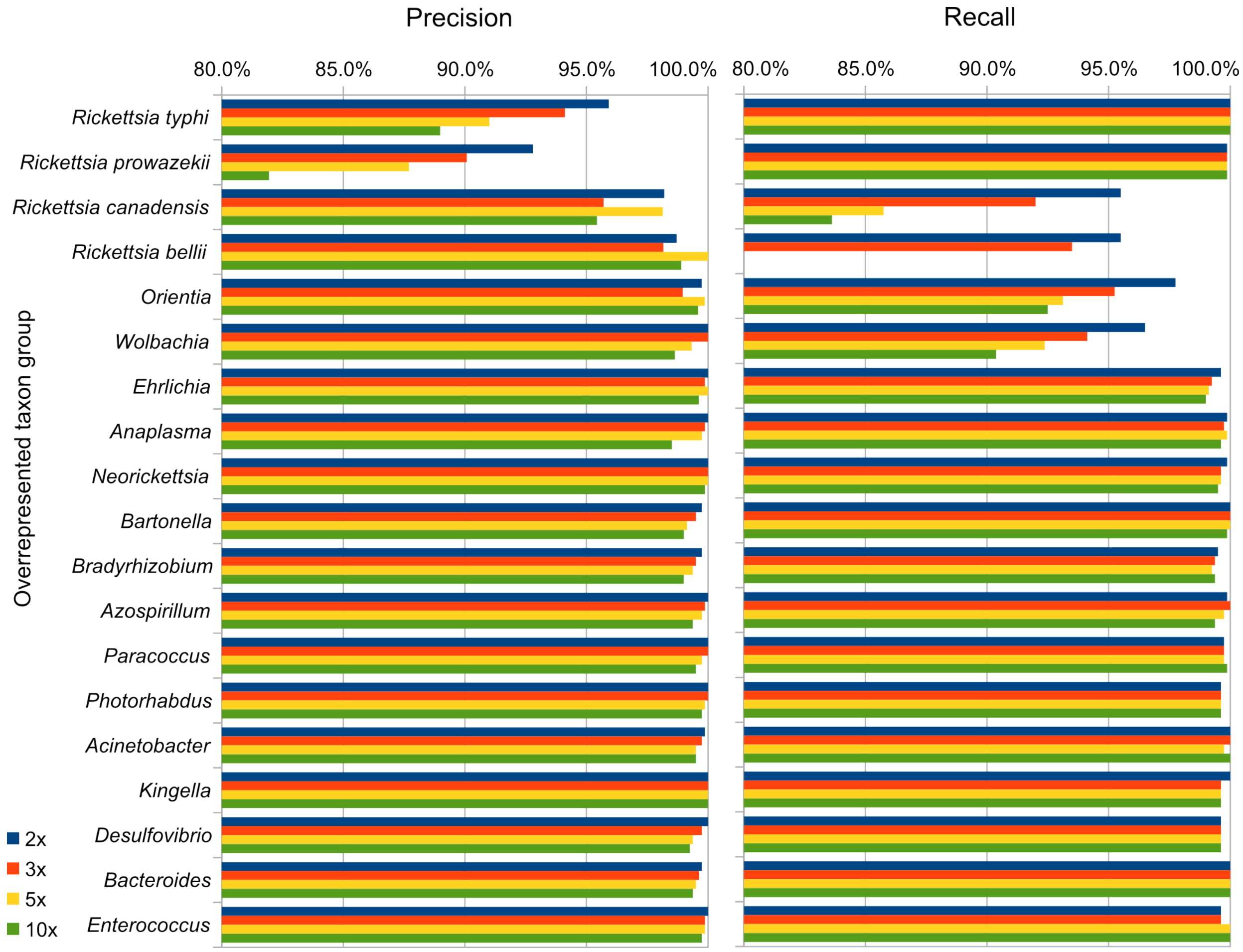


Figure S9 Stability of results on the *Rickettsia* dataset with simulated taxon sampling bias.

BLAST hits belonging to selected organisms (y-axis) were replicated into 2 (blue), 3 (red), 5 (yellow) and 10 (green) copies to simulate taxon sampling bias. Precision (left panel) and recall (right panel) of the result of HGTector analysis under the conservative criterion were computed with the standard result as the reference. The values of the invisible yellow and green bars in the right panel of *R. bellii* are 33.0% and 33.6%, respectively.

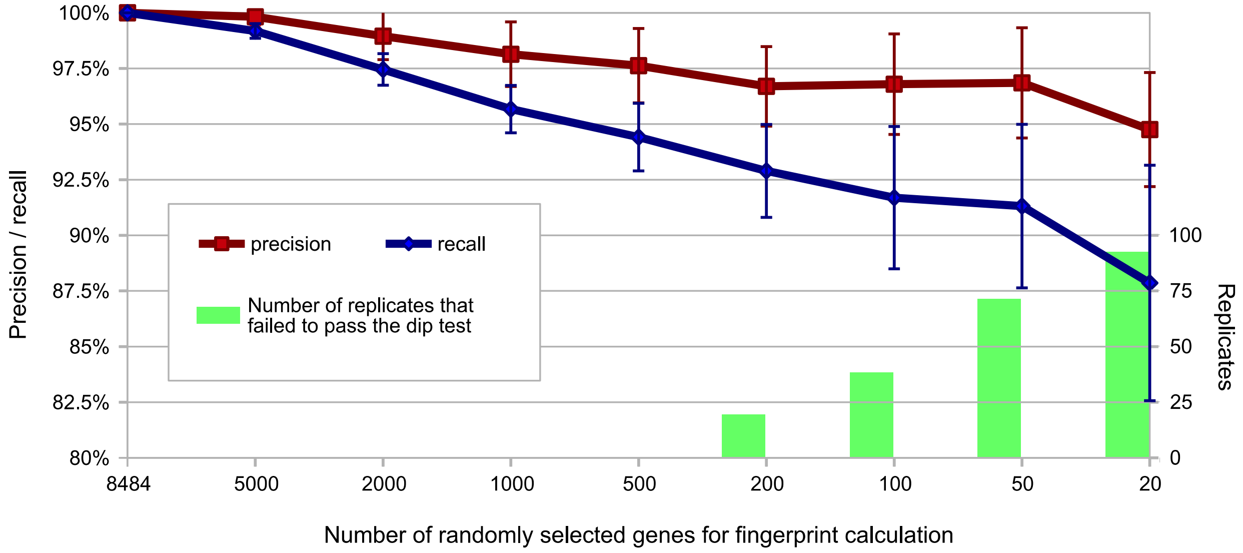


Figure S10 Stability of results on the *Rickettsia* dataset with smaller sample size of genes for fingerprint calculation.

Prediction results based on fingerprints of a randomly selected subset (x-axis) of input genes are plotted. Precision (red) and recall (blue) were computed referring to the standard result based on all (8484) genes. Each group has 100 replicates. Error bars indicate standard deviations. The number of replicates in each group that failed to pass the Hartigans' dip test is indicated by a green bar. These replicates were excluded from the precision / recall calculation.

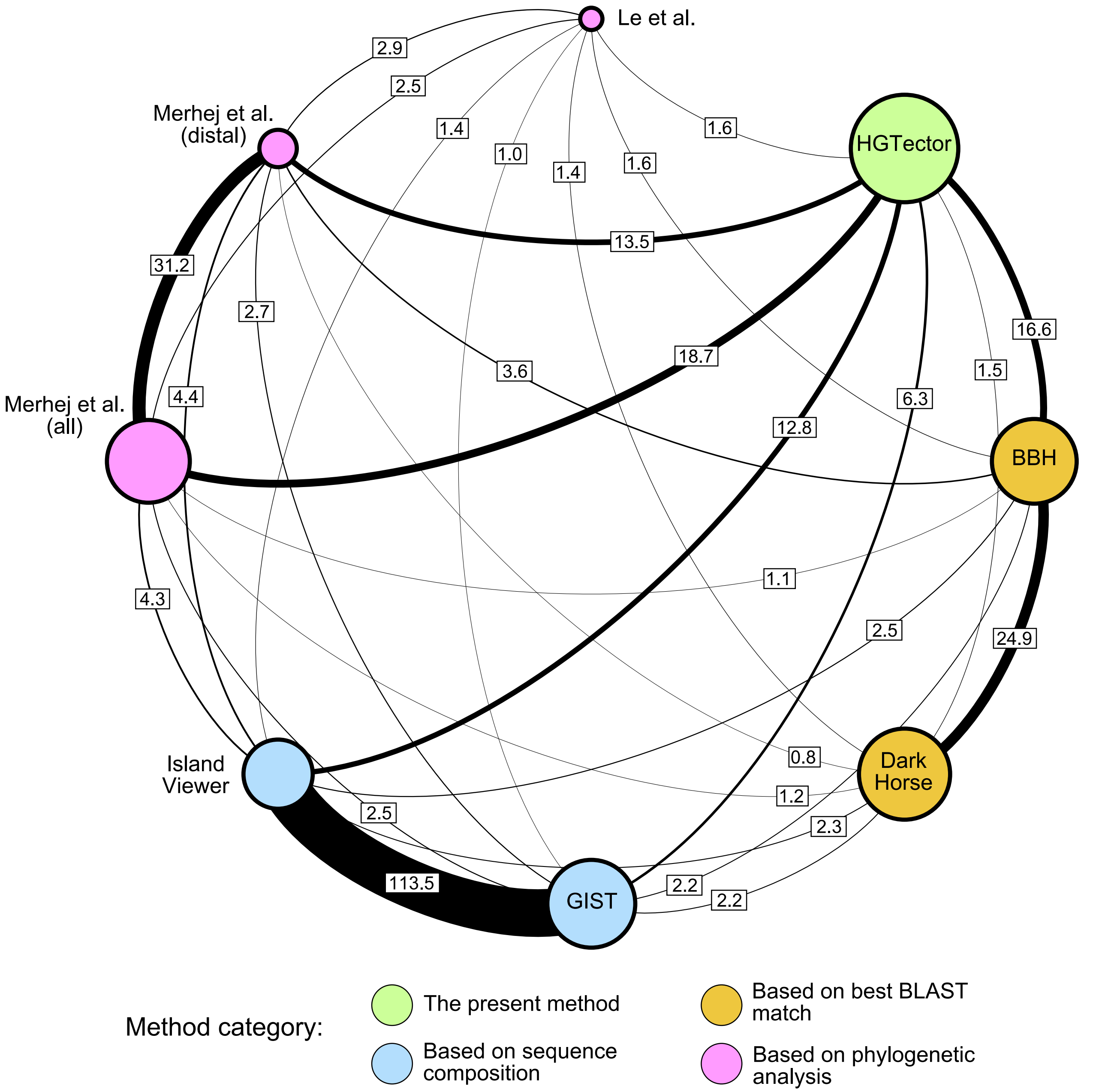


Figure S11 Comparison of prediction results in the *R. felis* genome by multiple methods.

An illustration of data provided in Table 3. Each method is indicated by a circle. Methods belonging to the same category are adjacent and in the same color. The area of a circle is directly proportional to the number of HGT-derived genes predicted by the method. The degrees of overlap between pairs of methods are represented by a circular network. The width of an edge connecting two circles is directly proportional to the overlap factor (OF, indicated in box) between the two methods. Therefore, the boldness of edges is a measurement of pairwise consistency between methods.
